# The Impact of Polymer Grafting from a Graphene Oxide Surface on Its Compatibility with a PDMS Matrix and the Light-Induced Actuation of the Composites

**DOI:** 10.3390/polym9070264

**Published:** 2017-07-03

**Authors:** Josef Osicka, Markéta Ilčíková, Miroslav Mrlik, Antonín Minařík, Vladimir Pavlinek, Jaroslav Mosnáček

**Affiliations:** 1 Centre of Polymer Systems, University Institute, Tomas Bata University in Zlin, Trida T. Bati 5678, 760 01 Zlin, Czech Republic; Josef.osicka@gmail.com (J.O.); mrlik@ft.utb.cz (M.M.); minarik@ft.utb.cz (A.M.); vladimir.pavlinek@5m.cz (V.P.); 2 Polymer Institute, Slovak Academy of Sciences, Dubravska cesta 9, 845 41 Bratislava 45, Slovakia; upolmail@savba.sk

**Keywords:** smart polymers, grafting method, reversible deactivation radical polymerization

## Abstract

Poly(dimethyl siloxane) (PDMS)-based materials with improved photoactuation properties were prepared by the incorporation of polymer-grafted graphene oxide particles. The modification of the graphene oxide (GO) surface was achieved via a surface initiated atom transfer radical polymerization (SI ATRP) of methyl methacrylate and butyl methacrylate. The modification was confirmed by thermogravimetric analysis, infrared spectroscopy and electron microscopy. The GO surface reduction during the SI ATRP was investigated using Raman spectroscopy and conductivity measurements. Contact angle measurements, dielectric spectroscopy and dynamic mechanical analyses were used to investigate the compatibility of the GO filler with the PDMS matrix and the influence of the GO surface modification on its physical properties and the interactions with the matrix. Finally, the thermal conductivity and photoactuation properties of the PDMS matrix and composites were compared. The incorporation of GO with grafted polymer chains, especially poly(*n*-butyl methacrylate), into the PDMS matrix improved the compatibility of the GO filler with the matrix, increased the energy dissipation due to the improved flexibility of the PDMS chains, enhanced the damping behavior and increased the thermal conductivity. All the changes in the properties positively affected the photoactuation behavior of the PDMS composites containing polymer-grafted GO.

## 1. Introduction

Photomechanical actuators are materials that convert photons into mechanical motion. In rubbery elastomeric matrices, stress and displacement are generated upon irradiation of a pre-strained sample via an external light source, i.e., the thermoplastic effect. The material absorbs the energy that is transported via thermal convection to the pre-strained polymer chains that contract and generate mechanical motion. The proposed mechanism has been studied in-depth by Cohn et al. [1]. Elastomeric photomechanical actuators are lightweight and remotely controlled. They are utilized as tactile devices [2,3], artificial muscles [4], vascular stents [5], intravascular embolic coils [6], micro-grippers [7], and micro-motors [8]. Liquid crystalline elastomers (LCE) [9,10], thermoplastic elastomers [5,11,12,13,14,15] (TPE) and cross-linked elastomers [6,16] have been reported as matrices for polymer composite-based photomechanical actuators. The negligible role of the host polymer on the actuation mechanism was revealed [16]. Poly(dimethyl siloxanes) (PDMS) are cross-linked elastomers that provide shape stability over time, biocompatibility and a variety of mechanical properties depending on the cross-linking density. Despite all their advantages compared to thermoplastic elastomers, their main drawback is that they are not re-processable.

The photomechanical actuation response can be enhanced via the utilization of additives or functional fillers, such as carbon nanotubes and graphene oxide [17,18,19], graphene [15,18], carbon black [18], and molybdenum disulfide [20,21]. The fillers can facilitate the absorption of light energy and its transfer through a material to enhance the actuation response. The actuation response can be affected by the type of filler, the concentration and the arrangement. To maximize its efficacy, the filler should be well-dispersed in the matrix [18,22]. An arrangement in the actuation direction has been reported to promote the actuation as well [9]. Regarding the shape of the fillers, the effectiveness increases with the dimensionality, which facilitates the orientation of the polymer chains; i.e., one dimensional carbon nanotubes were more effective than carbon black, but not as effective as two dimensional graphene platelets [18], and this was ascribed to the better dispersion in PDMS. Surprisingly, graphite oxide flakes did not exhibit a good performance.

Nevertheless, this drawback can be overcome using a suitable surface modification. The oxidized form of graphene, i.e., graphene oxide (GO), is rich in oxygen-containing functional groups that facilitate its further functionalization, and after reduction, it is electrically conductive [23]. Grafting polymer chains onto the surface of inorganic materials is a powerful approach to finely tune the surface chemistry of the particles. This approach enables control of the physicochemical properties, interfaces, and/or biochemical functionalities [24]. In addition, living/controlled polymerizations allow for tailoring of the molecular architecture of polymer brushes, e.g., the polymer chain uniformity (low dispersity), chain composition (homopolymers, block copolymers, gradient, periodic, and statistical), functionality and grafting density (sparse or dense).

Our previous studies revealed that the suitable surface modification of carbon nanotubes (CNTs) can effectively tailor the photoactuation response through the selective location of CNTs in block copolymers exhibiting thermoplastic elastomer properties [14,22,25,26]. Here, GO prepared via the oxidation of expanded graphite was investigated instead of CNTs. The amount and price of the prepared GO are more acceptable for large-scale applications. The hydroxyl and carboxylic groups enabled simple immobilization of the initiators for subsequent atom transfer radical polymerization (ATRP) from the GO surface. The polymer brushes of poly(methyl methacrylate) (PMMA) and poly(*n*-butyl methacrylate) (PBMA), which have comparable molar masses and grafting densities, were grafted from GO. The study focused on the effect of polymer modification on GO surfaces on the photoactuation response of the PDMS composites. The surface modification of GO by methacrylate polymers affected the compatibility with the PDMS matrix and the physical and thermal properties of the PDMS composites. Because of this effect, a polymeric material with high and reversible response to light was obtained.

## 2. Materials and Methods 

### 2.1. Materials

Graphite (powder, <20 μm, synthetic), sulfuric acid (H_2_SO_4_, reagent grade, 95–98%), sodium nitrate (NaNO_3_, ACS reagent, ≥99%), potassium permanganate (KMnO_4_, 97%) and hydrogen peroxide (H_2_O_2_, ACS reagent, 29.0–32.0 wt % H_2_O_2_ basis) were used as the chemical reagents for the proper exfoliation conditions to form the GO sheets. α-Bromoisobutyryl bromide (BiBB, 98%) served as an initiator and was linked onto the GO surface. The initiator bonding was performed in the presence of a proton scavenger, triethyleneamine (TEA, ≥99%). Methyl methacrylate (MMA, 99%), *n*-butyl methacrylate (BMA, 99%), ethyl α-bromoisobutyrate (EBiB, 98%), *N*,*N*,*N*′,*N*″,*N*″-pentamethyldiethylenetriamine (PMDETA, ≥99%), copper bromide (CuBr, ≥99%) and anisole (99%) were used as the monomer, initiator, ligand, catalyst and solvent, respectively. Diethyl ether (ACS reagent, anhydrous, ≥99%) was used as the drying agent. All the above listed chemicals were purchased from Sigma Aldrich (St. Louis, MO, USA) and all, except the MMA and BMA monomers, were used without further purification. MMA and BMA were purified by passing them through a neutral alumina column to remove the MEHQ inhibitor prior to their use. Tetrahydrofurane (THF, p.a.), acetone (p.a.), dimethylformamide (DMF, p.a.) and hydrochloric acid (HCl, 35%, p.a.) were obtained from Penta Labs (Prague, Czech Republic). Poly(dimethyl siloxane) (PDMS) Sylgard 184 was purchased from Sylgard, MI, USA and was used as received. Deionized water (DW) was used for all the experimental processes and washing routines.

### 2.2. Graphene Oxide Preparation and Initiator Immobilization

Graphene oxide (GO) was prepared from a graphite powder using a modified Hummers method [27]. The raw graphite (5 g) was vigorously stirred with H_2_SO_4_ (100 mL), and the mixture was cooled to 5 °C using an ice/water bath. Subsequently, NaNO_3_ (2.5 g) and KMnO_4_ (15 g) were gradually added. The mixture was stirred for an additional 6 h, and then, DW (300 mL) was added dropwise, while the temperature was kept below 40 °C. Finally, concentrated H_2_O_2_ (40 mL) was added, and the solution turned a brilliant brown, which indicated the complete oxidization of graphite. The product was separated in a high-speed centrifuge (Sorvall LYNX 4000, Thermo Scientific, Waltham, MA, USA) operating at 10,000 rpm for 20 min at 25 °C. The cleaning routine was based on the dispersion of GO in 0.1 M HCl and re-separation in a centrifugal field. The procedure was repeated with DW several times until the pH reached a value of 7. Then, the particles were lyophilized to remove any residual water after the purification process. Finally, a brown powder was obtained. 

The presence of the reactive groups on the GO surface was used for the reaction with BiBB to immobilize the ATRP initiator on the GO surface and prepare GO-I using the method described elsewhere [28,29].

### 2.3. Grafting Polymer Chains from the GO Surface

The GO sheets modified with the ATRP initiator (0.75 g) were transferred into a Schlenk flask equipped with a gas inlet/outlet and a septum. The flask was evacuated and replaced with argon several times. BMA (17.5 mL, 110 mmol) or MMA (11.8 mL, 110 mmol), EBiB (0.162 mL, 1.1 mmol), PMDETA (0.92 mL, 4.4 mmol), and anisole (15 mL) were then added to the flask, and the flask was degassed using several freeze–pump–thaw cycles. The CuBr catalyst (0.156 g, 1.1 mmol) was quickly added under an argon flow into the frozen system. Anisole was used as the solvent at an amount of 50 vol %. The polymerization mixture was stirred at 60 °C for 2 h, and then, the polymerization was stopped by exposure to air. The product was filtered, washed with DMF (2 × 200 mL) and acetone (2 × 200 mL), and dried with diethyl ether (2 × 100 mL).

### 2.4. Composite Preparation

An elastomeric matrix was prepared by mixing PDMS, silicone oil and a curing agent in a volume ratio of 8:2:1. The matrix was filled either with neat GO or GO with grafted PMMA or PBMA chains and was properly homogenized using mechanical stirring for 30 min at room temperature. The concentrations of the fillers were 0.1, 0.5 and 1 vol %. The mixture was poured into a Teflon-lined mold and evacuated at 60 mbar to eliminate the presence of air bubbles. Then, the mold was placed into an oven for 2 h at 60 °C to fully cross-link the PDMS-based composites. It should be stated that same procedure was used for the GO-I particles; however, even after 48 h, the composites were not fully cross-linked probably due to the presence of 2-bromoisobutyryl groups on the GO surface, which could eliminated the function of the cross-linker.

### 2.5. General Characterizations

Proton nuclear magnetic resonance (^1^H NMR) spectra were recorded at 25 °C using an instrument (400 MHz VNMRS Varian, Tokyo, Japan) and deuterated chloroform (CDCl_3_) as the solvent. The molar mass (*M*_n_) and dispersity (*Đ*) of the polymer chains were investigated by gel permeation chromatography (GPC) using a GPC instrument (PL-GPC220, Agilent, Tokyo, Japan) equipped with GPC columns (Waters 515 pump, two PPS SDV 5 μm columns (diameter of 8 mm, length of 300 mm, 500 Å + 105 Å)) and a Waters 410 differential refractive index detector at 30 °C. The samples for NMR spectroscopy and GPC analyses were prepared by their dilution with CDCl_3_ and THF, respectively, followed by the purification process, in which they were passed through a neutral alumina column. The neat GO and GO with a grafted polymer layer were observed using a transmission electron microscope (TEM, JEM-2100Plus, Jeol, Tokyo, Japan). The samples for the TEM analysis were prepared by dispersing the particles in acetone using mechanical stirring for 5 and 2 min of sonication and dropping the resultant suspension onto a copper grid. Fourier transform infrared (FTIR) spectra (64 scans, resolution of 4 cm^−1^) were recorded on a Nicolet 6700 (Nicolet, Madison, WI, USA) within a wavenumber range of 4000–600 cm^−1^, and the ATR technique with a germanium crystal was employed. The spectra were recorded at room temperature. The Raman spectra (3 scans, resolution of 2 cm^−1^) were collected on a Nicolet DXR (Nicolet, Madison, WI, USA) using an excitation wavelength of 532 nm. The integration time was 30 s, and the laser power on the surface was set to 1 mW. The powders were compressed to form pellets (diameter of 13 mm, thickness of 1 mm) on a laboratory hydraulic press (Trystom Olomouc, H-62, Olomouc, Czech Republic). The pellets were used for electrical conductivity measurements, and a two–point method at room temperature was applied with the help of an electrometer (Keithley 6517B, Beaverton, OR, USA). The presented results are the average values from 10 independent measurements. The contact angle (CA) measurements were evaluated using the static sessile drop method on the pellets and were performed on a surface energy evaluation system equipped with a CCD camera (Advex Instruments, Brno, Czech Republic). A droplet (5 μL) of PDMS was carefully dropped onto the surface, and the CA value was recorded. The presented CA results are the average values from 10 independent measurements. To confirm that the contact angle results were not affected by the roughness of the GO surface of the investigated pellets, atomic force microscopy (AFM) was used to investigate the surface topography using an atomic force microscope (AFM), model Dimension ICON (Bruker, Billerica, MA, USA). Measurements were performed at a scan speed of 1 Hz with a resolution of 256 × 256 pixels in the tapping mode at room temperature in an air atmosphere. A silicone-nitride probe with a resonant frequency of (150 ± 50) kHz and a stiffness constant of 5 N/m (MPP-12120, Bruker, Billerica, MA, USA) was used. The image analysis and surface roughness (Sa) determination were performed using the program Gwyddion v.2.48 (Gwyddion, Brno, Czech Republic).

The thermal conductivity was measured via a one-side contact method using the TCi model (C-term technologies, Fredericton, NB, Canada). The viscoelastic properties of both the nanocomposite and pure polymer matrix were studied via dynamic mechanical analysis (DMA) in the tensile mode. All the measurements were performed in the linear viscoelastic region. The measurements were performed at 1 Hz in the temperature range from −150 to 150 °C. Dielectric spectroscopy in the temperature range from −150 to 100 °C and in the frequency range from 10^−1^ to 10^7^ Hz was used to investigate the polymer chain dynamics.

The glass transition process was evaluated using the activation energies calculated from the Arrhenius equation (Equation (1)) to see the effect of modification on the relaxation processes in the PDMS based composites.

(1) $$f_{\beta }=f_{\infty }\exp\left(\frac{E_{a}}{k_{B}T}\right)$$

where *E*_a_ is the activation energy, $f_{\infty }$ is the pre-exponential factor, *T* is the temperature in Kelvin, and *k*_B_ is the Boltzmann constant.

### 2.6. Photoactuation

The photoactuation ability of both the matrix and composite samples was investigated using a thermal mechanical analysis (TMA, Mettler Toledo, Langacher, Switzerland) similar to that previously published [14]. A red LED diode (Luxeon Rebell, Philips, Amsterdam, The Netherlands) was used for the irradiation. The irradiation was applied for 10 s at 627 nm with a 6 mW light source intensity under a 10% pre-strain of the samples. The maximum value of the actuation is characterized by a change in the sample length during the exposure to light, Δ*L* = *(L*_0_ *− L)*/*L*_0_, where *L_0_* is the length of the non-irradiated sample, and *L* is the length of the irradiated sample.

## 3. Results and Discussion

### 3.1. Synthesis of GO-Polymer-Modified Particles

**Figure 1:**
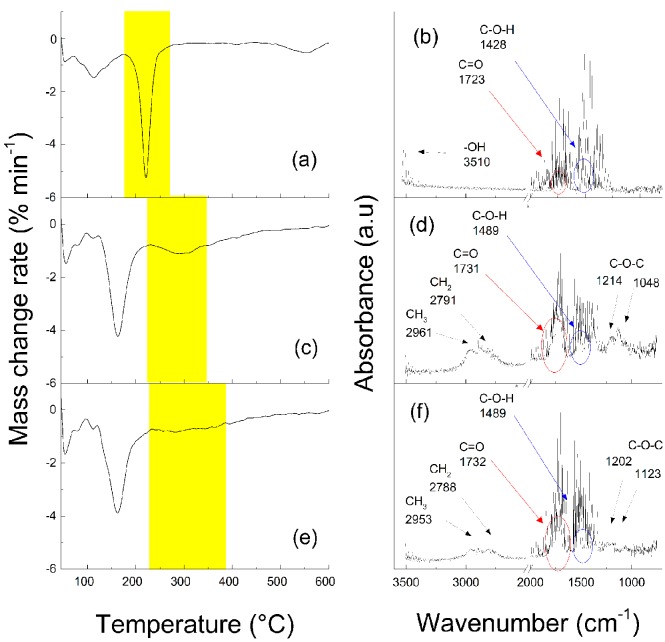
TGA analysis (**a**,**c**,**e**) with on-line monitoring of the Fourier transform infrared (FTIR) spectra (**b**,**d**,**f**) for neat graphene oxide (GO) (**a**,**b**), GO–poly(methyl methacrylate) (PMMA) (**c**,**d**) and GO–poly(*n*-butyl methacrylate) (PBMA) (**e**,**f**) particles.

The modification of the GO particles with PMMA (GO–PMMA) and PBMA (GO–PBMA) polymer chains was performed via surface initiated ATRP, as previously described for other methacrylate-based monomers [29]. The MMA and BMA conversions, which were calculated from the ^1^H NMR spectra, were 89% and 91%, respectively. Using a sacrificial initiator in addition to the initiator immobilized on the GO surface allowed for the determination of the molecular characteristics of the polymer chains with the consideration that the growth of both the free and GO surface-bonded polymer chains is comparable [30]. The molecular weight (*M*_n_) and polydispersity index (*Đ*) determined for PMMA were 5620 g mol^−1^ and 1.18, respectively, and they were 5210 g·mol^−1^ and 1.21, respectively, for PBMA. The molar masses correlated well with the monomer conversions. The successful grafting process was confirmed by the FTIR investigations with on-line monitoring during the TGA measurements. In Figure 1a, a release of oxygen-containing groups from the neat GO surface can be seen in the temperature range of 150–300 °C. In the same temperature range, the FTIR spectrum in Figure 1b of the released compound was analyzed, and the vibrations from the hydroxyl groups at 3510 cm^−1^, a small amount from the carbonyl groups at 1723 cm^−1^ and the stretching of the hydroxyl groups at 1423 cm^−1^ were found. The release of the groups from PMMA was observed in the TGA in the range of 220–380 °C, as seen in Figure 1c. The FTIR from the released groups showed an increase in the peaks of the carbonyl groups at approximately 1731 cm^−1^ in comparison to the neat GO, where this peak is almost negligible, and the appearance of peaks in the alkyl vibrations region of 2600–3000 cm^−1^ (Figure 1d), which confirmed the presence of PMMA on the GO surface. Similar results were obtained for the GO modified with the PBMA chains (Figure 1e,f), but the release of the groups from PBMA occurred at higher temperatures. To quantify the amounts of the individual components in the GO, GO–PMMA and GO–PBMA particles, the TGA data were analyzed. It was found that, similar to our previous studies [28,29], the amount of the oxygen-containing groups for the neat GO was app. 30%. On the other hand, for both cases of polymer modification, the amount of polymer on the hybrid particles was app. 9% for PMMA and 11% for PBMA, and the amount of oxygen-containing groups was nearly 21% for PMMA and 19% for PBMA, which indicated partial reduction of GO surface during its modification as observed also previously [28].

### 3.2. Transmission Electron Microscopy

**Figure 2:**
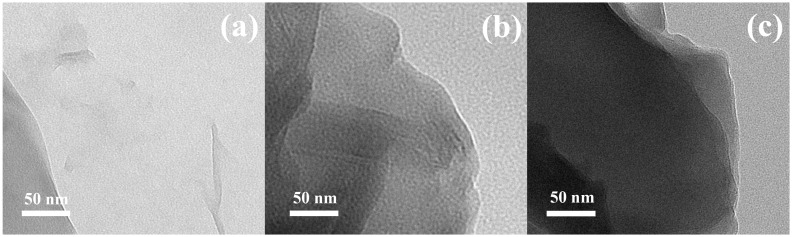
Transmission electron microscope (TEM) images of the neat GO (**a**), GO-PMMA (**b**) and GO–PBMA (**c**).

In Figure 2, the TEM images of all the investigated GO particles are shown. In the case of neat GO, the proper exfoliation of the graphite powder using the Hummers method was achieved, and the neat GO sheet can be clearly seen on the TEM image (Figure 2a). The sheet-like morphology was also observed in the case of the GO modified with the PMMA chains (Figure 2b) when the 2D shape was sustained on the same level. The slightly darker contrast of the GO–PMMA sheet is due to the presence of the polymer layer on the surface of the GO. In the case of the GO–PBMA particles (Figure 2c), the 2D shape was again observed, which indicated they had the same morphology as the neat GO and GO–PMMA. Here, several layers of GO most likely lay on top of each other, and with the sustainable polymer layer of PBMA on the surface of the GO, they provide the higher contrast of the image. The sharp edges of the neat GO become smoother in the case of both the modified GOs due to presence of polymer chains, which again confirmed the successful modification of the GO surface.

### 3.3. Reduction of the GO Particles during the Synthesis

**Figure 3:**
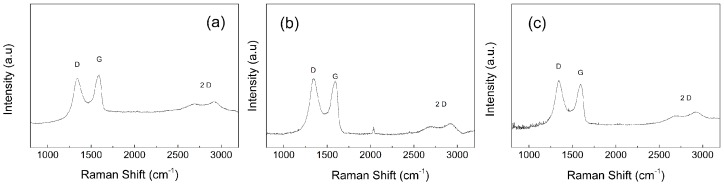
Raman spectra of the neat GO (**a**), GO–PMMA (**b**) and GO–PBMA (**c**).

In our previous work, we showed that the reduction of GO can occur during SI ATRP [28]. To confirm the reduction of the GO particles during the SI ATRP process, Raman spectroscopy was used as a useful tool to compare the broad D and G peaks corresponding to the sp^2^ and sp^3^ hybridized forms of the carbon atoms in the GO sheets. In Figure 3, a comparison among the neat GO sheets, GO–PMMA and GO–PBMA can be seen. A significant reduction in the GO sheets was achieved, and it can be observed from the significant change in the D/G peak intensity ratio (*I*_D_/*I*_G_) obtained after the SI ATRP process. Thus, a change from 0.90 to 1.05 and 1.08 was observed after the SI ATRP of MMA and BMA, respectively. It can also be seen that the 2D structure of GO created during the oxidation was nearly the same after modification with both the PMMA and PBMA brushes. The conductivity measurements confirmed the results obtained from the Raman spectroscopy, which indicated the reduction of GO after modification. The conductivity slightly increased from 1.2 × 10^−8^ S cm^−1^ for the neat GO to 6.3 × 10^−8^ S cm^−1^ for the GO–PMMA and 2.1 × 10^−7^ S cm^−1^ for the GO–PBMA. Therefore, it can be stated that within the SI ATRP process, a partial reduction of the GO with the simultaneous grafting of PBA polymer brushes was achieved.

### 3.4. Compatibility of the Particles with the PDMS Matrix

**Figure 4:**
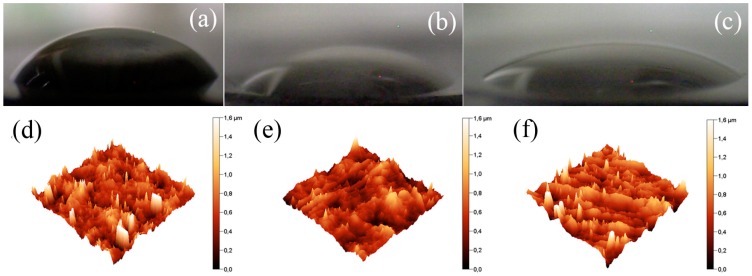
Images from the CCD camera of the 5 µL PDMS droplets on the neat GO (**a**), GO–PMMA (**b**) and GO–PBMA (**c**), and the atomic force microscopy (AFM) investigations of the surface roughness for the (**d**) neat GO; (**e**) GO–PMMA and (**f**) GO–PBMA.

The compatibility of the GO particles with the surrounding matrix when they are dispersed is a crucial part of their potential applications as light sensors or actuators [14,25]. Therefore, the compatibility between the neat GO or polymer-functionalized GO and PDMS was investigated via contact angle measurements. As seen in Figure 4a, the contact angle between the neat GO pellet and the PDMS droplet was 49.9° ± 3.2°, which indicated a relatively poor compatibility. The presence of the short polymer chains of PMMA improved the compatibility and decreased the contact angle to 38.7° ± 2.7° (Figure 4b). In addition, the longer aliphatic butyl chain present in PBMA decreased the contact angle to 28.7° ± 2.7° (Figure 4c). This result indicated significantly improved interactions between PDMS and the surface of the GO–PBMA in comparison with the surface of neat GO, which contains only hydroxyl, carbonyl, carboxyl or epoxy groups. To prove that these results were not affected by the surface roughness, an AFM investigation was performed. As seen in Figure 4d–f, the roughnesses were nearly identical for all the investigated samples, and the surface roughness (Sa) was found to be 154, 143 and 162 nm for GO, GO–PMMA and GO–PBMA, respectively. Therefore, it can be concluded that the contact angle was significantly affected by the chemical modification (coating) and that the roughness played a negligible role.

### 3.5. Dielectric Investigation of the Polymer Chain Dynamics

**Figure 5:**
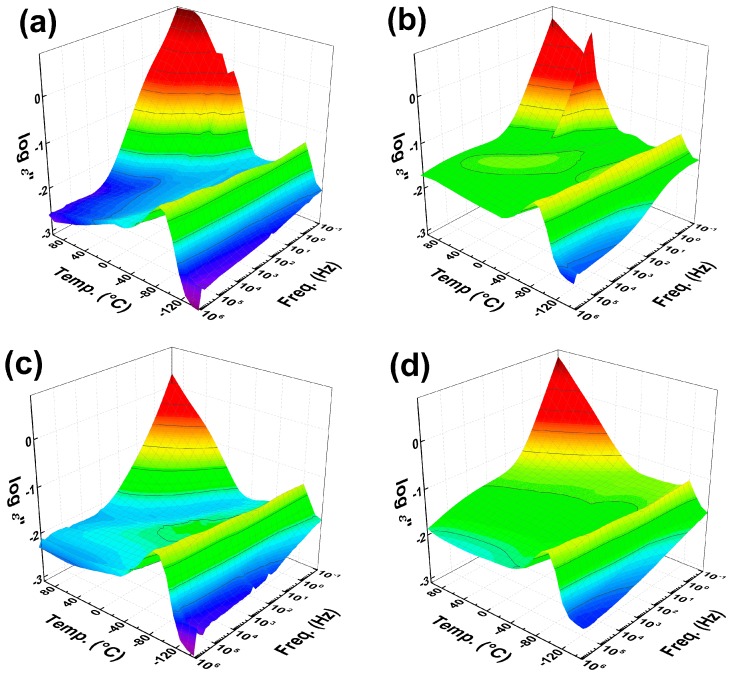
3D plots of dielectric properties for the neat PDMS matrix (**a**) and GO/PDMS composites with 0.5 vol % of GO (**b**), GO–PMMA (**c**) and GO–PBMA (**d**) particles.

To investigate the influence of the GO surface modification on the polymer chain dynamics, dielectric spectroscopy measurements were performed. As seen in Figure 5, the presence of the neat GO in the PDMS matrix slightly affected the response of the PDMS polymer chains; however, in the case of GO–PMMA and GO–PBMA, the presence of short polymer grafts significantly shifted the glass transition temperature to lower values. To confirm this change, the activation energies of the glass transition process were calculated, and the effects of the filler nature and its loading were investigated. Within the range of the studied frequencies, a linear dependence of the *T*_g_ on the frequency was observed; therefore, a simple Arrhenius equation was used for the determination of the glass transition activation energies. The values are summarized in Table 1. The activation energy decreased with the increasing amount of filler. This indicated that the filler behaves as a softener in this case and makes the transition easier, and the overall movement of the main PDMS backbone requires less exertion. Moreover, from the results, it can be clearly seen that the longer pendant aliphatic chain in PBMA caused a more significant softening of the polymer backbone, and, thus, the PDMS chains are more movable. This behavior should have a positive impact on the investigated photoactuation performance.

### 3.6. Dynamic Mechanical Analysis

**Figure 6:**
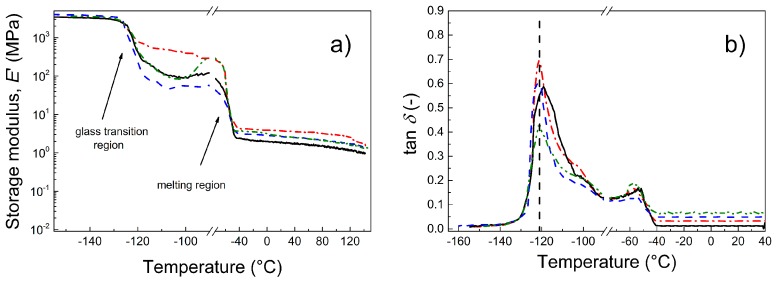
Dependence of the storage modulus (**a**) and tan δ (**b**) for a broad temperature range for neat PDMS (black solid line) and for PDMS composites containing 0.1 vol % of neat GO (red dash dot line), GO–PMMA (blue dashed line) and GO–PBMA (green dash-dot-dot line).

Because photoactuation is a dynamic process, a dynamic mechanical analysis of the prepared composites is crucial to investigate their suitability for intended applications. Through this investigation, the interactions between the particles and the matrix can also be estimated. As seen in Figure 6, below the glass transition temperature, *T*_g_, all the composites exhibited nearly the same mechanical properties (Figure 6a). However, due to the presence of various fillers, the *T*_g_ of the composites slightly changed. Moreover, another transition region connected to the melting of the small crystalline phase present in PDMS was observed, which was also observed elsewhere [31]. Only a minor effect of the fillers was seen in the melting temperature region. In the region of utilization, the storage moduli were nearly independent of the temperature up to 40 °C. In this case, the best mechanical performance was observed for the neat GO. The reason for this could be possible covalent bonding between the OH groups and the PDMS polymer chains, which was observed by Bose et al. for OH groups of carbonyl iron and PDMS [32]. After coating of GO with the PMMA or PBMA chains, the partial physical entanglement provided an improved compatibility between PDMS and GO–PMMA or GO–PBMA compared with neat GO. However, at the same time, the short polymer chains at the GO surface can serve as plasticizers and thus the final PDMS material can be more flexible; but the PDMS was still reinforced in comparison to the neat PDMS matrix. The damping properties of the PDMS-containing polymer-grafted GO were slightly enhanced, as indicated by the higher tan delta values (Figure 6b). This is highly desirable from a photoactuation performance point of view because physical entanglements and GO–PBMA can provide the best flexibility in the prepared composite. In fact, this is in good agreement with the activation energies determined from the dielectric spectroscopy and will be confirmed by the photoactuation investigation (see below).

**Figure 7:**
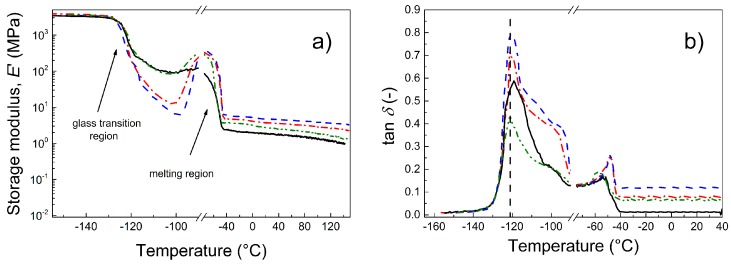
Dependence of the storage modulus (**a**) and tan δ (**b**) for a broad temperature range for neat PDMS (black solid line) and for PDMS composites containing various concentrations of GO–PBMA: 0.1 vol % (green dash-dot-dot line), 0.5 vol % (red dash dot line) and 1 vol % (blue dashed line).

To investigate the effect of the filler loading on the mechanical performance, the composites with the most promising properties, i.e., those containing GO–PBMA particles with various filler loadings (0.1, 0.5 and 1 vol %), were investigated (Figure 7). It can be clearly seen that below the *T*_g_, the materials exhibited nearly the same behavior. Over the *T*_g_, the samples with a higher filler loading (0.5 and 1 vol %) exhibited a significant drop in the storage moduli (Figure 7a) with a subsequent increase to a maximum before melting. The drop can be caused by interactions between the GO-PBMA particles and the polymer matrix, which make the PDMS matrix more flexible, as described above. This behavior can also be connected to the decreased activation energy, which is in good agreement with our previous observations. After the melting of the crystalline phase, the present GO–PBMA particles still act as a reinforcing filler from a storage modulus point of view. The *T*_g_ slightly shifted to a lower temperature for all the filler contents (Figure 7b), which indicated the intervention of the GO–PBMA particles in the glass transition process. The right shoulder is most likely connected to the physical interactions of the polymer chains of GO–PBMA with the PDMS matrix, which were already described in Figure 7a. Moreover, the damping behavior of the composites was enhanced with an increase in the filler content, which indicated the relatively high energy dissipation caused by the more flexible structure present in the GO–PBMA/PDMS composites. It can be concluded that the modification of GO with various polymers can affect the mechanical performance; i.e., the storage moduli can be tailored using various modifications, and the polymer modification of GO particles in PDMS composites significantly contributes to the increasing energy dissipation due to the improved flexibility and provides an enhanced damping behavior.

### 3.7. Thermal Conductivity

According to a previous study [19], the thermal conductivity is another crucial parameter affecting the photoactuation performance. As seen in Table 2, the pure PDMS matrix has a thermal conductivity of 0.071 W mK^−1^, and it increased with the increasing amount of filler for all the investigated samples. Due to the improved compatibility with the PMMA and PBMA polymer chains attached on the GO surface, these samples exhibited improved thermal conductivities compared to the neat GO. The best thermal conductivity was found for the composites containing the GO–PBMA particles. Thus, these composites can provide a significant contribution to enhanced photoactuation capabilities due to the significantly improved heat distribution within the samples.

### 3.8. Photoactuation Performance

**Figure 8:**
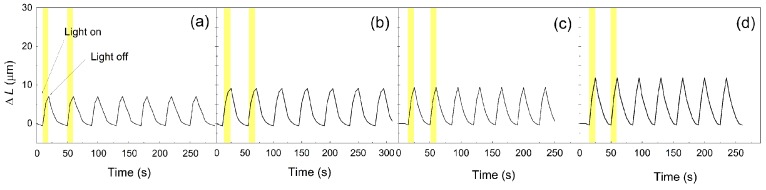
Photoactuation performance of the pure PDMS (**a**) and the PDMS composites containing 0.1 vol % of neat GO (**b**), GO–PMMA (**c**) and GO–PBMA (**d**).

The photoactuation performance was investigated for various composite compositions, and the impact of the filler modification and the filler loading on the final properties was elucidated. As seen in Figure 8, the pure matrix showed some actuation performance. The determined maximum value of actuation, Δ*L*, was 7.1 μm, and the recovery time was 30 s. The addition of 0.1 vol % of neat GO particles increased the maximum value of the actuation to 9.1 μm, and this was likely due to the improved heat transfer that was observed during the thermal conductivity measurement. However, the recovery time was the same as that for the pure PDMS matrix. In the case of the GO–PMMA-based composite, the maximum actuation increased to 9.4 μm, and the recovery time was shortened to 25 s. A similar behavior was obtained for the sample containing GO–PBMA particles. The actuation performance was 11.8 μm, and the recovery time similar to that of the GO–PMMA composites was achieved. The main reason for this capability is the proper incorporation of the GO–PMMA and GO–PBMA particles into the PDMS matrix, which causes better shape recovery and improved heat transitions within the samples.

**Figure 9:**
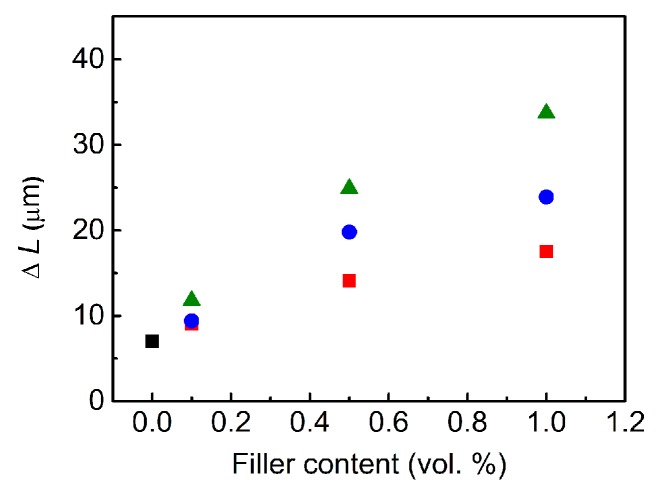
Dependence of the filler content on the change in the length for the pure PDMS matrix (black squares) and PDMS composites containing neat GO (red squares), GO–PMMA (blue circles) and GO–PBMA (green triangles). Error bars are not higher than the size of the symbols.

The impact of the filler content on the photoactuation performance of the PDMS materials was investigated, and the results are plotted in Figure 9. In all cases, the Δ*L* increased with an increasing amount of filler. The best capability was found for the composites containing the GO–PBMA particles, and the maximum actuation value for a composite containing 1 vol % of GO–PBMA was approximately 5 times higher than that for pure PDMS. The main reason for this significant improvement was already proposed by previous investigations and is likely the enhanced flexibility of the polymer composite, which was confirmed by the lower activation energy, higher damping and enhanced thermal conductivity, improving the heat distribution within the composite samples. The polymer grafting from GO approach for the preparation of composites with photoactuation capabilities provides a system with an enhanced performance in comparison to other systems, including 2 wt % of neat CNTs, carbon black, GO or graphene nanoplatelets in the PDMS composites. The systems mentioned last showed a relative change in the length, below 25 µm, when recalculated for our conditions [18].

## 4. Conclusions

In summary, this study investigated the influence of grafting polymer chains from a GO surface on the properties of the resulting PDMS-based composites. Short PMMA or PBMA polymer chains were grafted from the GO surface via SI-ATRP, and a slight reduction of the GO surface during the grafting was determined using Raman spectroscopy and conductivity measurements. The contact angle measurements confirmed the improved compatibility of the polymer-grafted GO particles with the PDMS matrix, especially when the GO was grafted with the PBMA chains. The polymer chain dynamics were investigated using dielectric spectroscopy, and the Arrhenius equation was applied to calculate the activation energy of the relaxation around the glass transition temperature. It was found that the modifications caused a significant decrease in the activation energy and acted as a plasticizer for the PDMS matrix, which has a positive influence on the photoactuation behavior. This result was confirmed by the dynamic mechanical analysis.

The thermal conductivity was also improved by the presence of the GO particles, and the polymer-grafted GO more significantly enhanced the thermal conductivity due to the better dispersibility of the particles in the PDMS matrix. Finally, the photoactuation performance was elucidated, and it was found that the composites including the GO–PBMA particles had the best capability due to the more flexible polymer chains and better heat distribution within the PDMS matrix.

## Figures and Tables

**Table 1:** Activation energies of the glass transition process for the pure PDMS and PDMS composites with various filler loadings.

Sample Code	Activation Energy (kJ·mol^−1^)
pure PDMS	45.70
0.1 vol % neat GO PDMS	36.57
0.5 vol % neat GO PDMS	34.97
1 vol % neat GO PDMS	20.35
0.1 vol % GO–PMMA PDMS	35.99
0.5 vol % GO–PMMA PDMS	23.58
1 vol % GO–PMMA PDMS	10.16
0.1 vol % GO–PBMA PDMS	25.39
0.5 vol % GO–PBMA PDMS	22.79
1 vol % GO–PBMA PDMS	9.49

**Table 2:** Thermal conductivity of pure PDMS and PDMS composites with various filler loadings.

Sample Code	Thermal Conductivity (W·mK^−1^)
pure PDMS	0.071
0.1 vol % neat GO/PDMS	0.144
0.5 vol % neat GO/PDMS	0.156
1 vol % neat GO/PDMS	0.161
0.1 vol % GO–PMMA/PDMS	0.153
0.5 vol % GO–PMMA/PDMS	0.174
1 vol % GO–PMMA/PDMS	0.209
0.1 vol % GO–PBMA/PDMS	0.151
0.5 vol % GO–PBMA/PDMS	0.186
1 vol % GO–PBMA/PDMS	0.250
